# Supplementary material for: Knowledge, attitude, and uptake of human papilloma virus vaccine and associated factors among female preparatory school students in Bahir Dar City, Amhara Region, Ethiopia
Source: PLoS One. 2022 Nov 21;17(11):e0276465. doi: 10.1371/journal.pone.0276465 (PMC9678319; doi:10.1371/journal.pone.0276465)
Supplement: S1 File — (DOCX) [file pone.0276465.s001.docx]

#
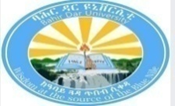


# English version verbal Consent Form

**Bahirdar University**

**College of Medicine and health sciences School of public health Department of Reproductive health**

**Consent form**

This questionnaire develops to assess knowledge, attitude, and uptake of Human Papilloma Virus (HPV) vaccine among female preparatory school students in Bahir Dar city, Ethiopia,2021

My name is ______________ I am collecting data for Ms. Etenesh Adela who is postgraduate student with an MSc in Reproductive Health. I have given my consent to participate in the study entitled as “knowledge, attitude, and uptake of Human Papilloma Virus (HPV) vaccine among at preparatory school female in Bahir Dar city, Ethiopia.” I brought these questionnaires to ask you a few questions about knowledge, attitude and uptake of Human Papilloma Virus (HPV) vaccine. This will help us to identify the main risk factors for knowledge, attitude of HPV vaccine based on your answers. Your name will not be written in this form and will never be used regarding with any information that you tell us. All information that is given by you will be kept strictly confidential. Your participation in this study is entirely voluntarily and you are not obliged to answer any question that you do not wish to answer. If you fill discomfort with the interview, please fill free to drop it any time you want. This interview will take about 25 minutes.

**Are you willing to take part in the study Yes No**

1. If yes, continue to the next page. If no, skip to the next participant by writing reasons for his/ her refusal.

1. Investigator’s name Etenesh Adela Lakneh Sign ________

2. Data collector Name __________sign--------

3**.** Supervisor name ___________Sig. _____

- Date of data collection _/__/_

## English version information sheet

Name of the investigator: Etenesh Adela Lakneh

**Name of organization**: Bahir Dar University College of Medicine and Health Science School of Public Health

**Sponsor**: Amhara Regional health bureau office

**Title of the study**: knowledge, attitude, and uptake of Human Papilloma Virus (HPV) vaccine among preparatory school female students in Bahir Dar city, Ethiopia

##

## Annex I: English version questionnaire

Quantitative study questionnaire

01. Code number _____

02. Date of data collection _____/_____/_____ E.C

03. Data collector name _______________ Signature __________

Encircle the answer for multiple choice questions and fill your answer for blank spaces Annex I: English version questionnaire

Encircle the answer for multiple choice questions and fill your answer for blank spaces

| **Part I. Socio-demographic related question** | | | **Code** |
| --- | --- | --- | --- |
| No | Question | Response |  |
| 101 | Age | _______ years |  |
| 102 | Childhood residence | 1.Rural  2.Urban |  |
| 103 | Marital status | 1.Single  2.Married  3.Divorced  4.Widowed |  |
| 104 | Religion | 1.Orthodox  2.Muslim  3.Catholic  4.Protestant  5.others (specify) _________ |  |
| 105 | Ethnicity | 1.Amahara  2 Oromo  3.Tigry  4.Other (specify)_________ |  |
| 106 | Your grade level | 1.Grade 11  2.Grade 12 |  |
| 107 | In which type of institution did you complete your elementary school? | 1.Public school  2.Private school |  |
| 108 | Where is your current school? | 1.Public school  2.Private school |  |
| 109 | Your father's level of education? | 1.Unable to read and write  2. Informal education (can read and write)  3. Primary (1-8)  4. Secondary (9-12)  5.Diploma and above  6. Others, specify------------ |  |
| 110 | Your mother’s level of education? | 1.Unable to read and write  2.Informal education (can read and write)  3. Primary (1-8)  4. Secondary (9-12)  5.Diploma and above |  |
| 111 | Your mother’s occupation status | 1. Housewife 2. Self-employees 3. Government employees 4. Non-governmental employees 5. Farmer 6. Merchant 7. Others, specify------------ |  |
| 112 | Average family monthly income? | 1. ---------birr |  |
| **Part II: Reproductive health related questions** | | |  |
| **201** | Have you ever had sexual intercourse? | 1.Yes  2.No |  |
| 202 | How many sexual partners did you have? | -------(number) |  |
| 203 | Have you discussed sexual health issues with your partners? | 1 Yes  2.No |  |

| **Part Discourse of information** | | | |
| --- | --- | --- | --- |
| 301 | Have you heard about human papilloma virus? | 1.Yes  2.No | If your answer is no skip to 401 |
| 302 | If yes for “Q no 301”, what is your source of information? (Mentioned more than one multiple choice is possible) | 1.Newspapers  2.Radio /Television  3.Internet  4.Healthcare providers  5. School  6. Others…… |  |
| Part IV **Knowledge about HPV infection about preparatory school female students** | | | |
| 401 | Did you know that having multiple-sexual partner is the risk factor for human papilloma virus infection? | 1.Yes  2.No |  |
| 402 | Does sexual intercourse before marriage increases the risk of human papilloma virus infection? | 1.Yes  2.No |  |
| 403 | Is cigarette smoking increase the risk of human papilloma virus infection | 1.Yes  2.No |  |
| 404 | Is a human papillomavirus infection the cause of cervical cancer? | 1.Yes  2.No |  |
| 405 | Can a person transmit human papillomavirus to his or her partner even if he or she has no symptoms of infection? | 1.Yes  2.No |  |
| 406 | Can human papilloma virus infection be treated with antibiotics? | 1.Yes  2.No |  |
| 407 | Dose genital wart is caused by human papilloma virus infection? | 1.Yes  2.No |  |
| 408 | Most people with genital human papillomavirus infection do not have visible symptoms? | 1.Yes  2.No |  |
| 409 | Is vaginal douching after intercourse effective in preventing the human papillomavirus infection? | 1.Yes  2.No |  |
| 410 | Does Sexual contact is the transmitting route of human papilloma virus infection? | 1.Yes  2.No |  |

**Part V Source of information about HPV vaccine about preparatory school female students**

| 501 | Have you ever heard about human papilloma virus vaccine? | 1.Yes  2.No | If your answer is no skip 601 |
| --- | --- | --- | --- |
| 501.1 | If yes for “q no 401”, what is your source of information? (More than one multiple choice is possible) | 1.Newspapers  2.Radio / Television  3.Internet  4.Healthcare providers  5.School  6.Others (specify)…… |  |
| **Part VI. Knowledge about HPV vaccine about preparatory school female students** | | | |
| 601 | Does human papilloma virus vaccine prevent human papilloma virus infection? | 1.Yes  2.No  3.I don’ know |  |
| 602 | Does human papilloma virus vaccine prevent cervical cancer? | 1.Yes  2.No  3.I don’ know |  |
| 603 | Is the human papillomavirus vaccine used to prevent genital warts? | 1.Yes  2.No  3.I don’ know |  |
| 604 | Should a human papilloma virus vaccine be given prior to the first sexual intercourse? | 1.Yes  2.No  3.I don’ know |  |
| 605 | Can human papilloma virus vaccine be given to people who have had sex? | 1.Yes  2.No  3.I don’ know |  |
| 606 | Can human papillomavirus vaccine be given to a woman who is already infected with HPV? | 1.Yes  2.No  3.I don’ know |  |
| 607 | What age range is recommended for vaccination against human papillomavirus infection? | 1.________  2. I don’t know |  |
| 608 | How many doses are recommended for a human papillomavirus vaccine? | 1________  2. I don’t know |  |
| 609 | Did you know the schedule for a human papillomavirus vaccine? | 1.Yes  2. No  3.I don’ know |  |
| 610 | what is the site of administration of the vaccine? | --------------------------- |  |

**Part VII. Assessment of respondent’s attitude towards HPV vaccination using 12 statements with a 5-point Likert scale. Please mark “X” in each item for your answer.**

| s.no | Items | | Strongly agree (5) | | Agree (4) | Neutral (3) | Disagree (2) | | Strongly Disagree (1) |
| --- | --- | --- | --- | --- | --- | --- | --- | --- | --- |
| 701 | HPV vaccine is effective in preventing cervical cancer | |  | |  |  |  | |  |
| 702 | I will take the vaccine because I feel at risk of getting HPV | |  | |  |  |  | |  |
| 703 | Person who has only one sex partner can protect from HPV infection | |  | |  |  |  | |  |
| 704 | It's not necessary to get the human papilloma virus vaccination | |  | |  |  |  | |  |
| 705 | I believe that the side effects of the vaccine are reasonable and will not prevent me from taking the vaccine. | |  | |  |  |  | |  |
| 706 | I feel it is better to be vaccinated before becoming sexually active | |  | |  |  |  | |  |
| 707 | More information on HPV and its vaccine will be needed before I take the vaccine. | |  | |  |  |  | |  |
| 708 | Human papilloma virus vaccine may have long negative effect | |  | |  |  |  | |  |
| 709 | I feel only sexually active ladies should get the vaccine | |  | |  |  |  | |  |
| 710 | My parents would not allow me to get the vaccine. | |  | |  |  |  | |  |
| 711 | Education on HPV should be implemented at school | |  | |  |  |  | |  |
| 712 | HPV vaccination should be included on the National Program on immunization | |  | |  |  |  | |  |
| **Part VIII. Questions on HPV vaccine uptake** | | | | | | | | | |
| 801 | | Have you ever taken human papilloma virus vaccine? | | 1.Yes  2.No  3.I don’t remember | | | | If No skip to Q 806 | |
| 802 | | If yes answer Q 801 how many times did you take human papilloma virus vaccine? | | 1. One times  2.Two times  3. I don’t remember | | | |  | |
| 803 | | What is the interval between the given does? | | 1._______  2. I don’t remember | | | |  | |
| 804 | | At what age do you take a human papillomavirus vaccine? | | 1._______year | | | |  | |
| 805 | | Where did you take the vaccine for human papillomavirus? | | 1.Health institution  2.Shcool  3.Others______ | | | |  | |
| 806 | | If the answer to Q.801 is no, what is the reason why you did not take the vaccine? | | 1.I believed no need of human papilloma virus vaccine  2.Not informed by health care provider  3.I did not know about safety of the vaccine  4.I did not know where the vaccine is gotten  5. others specify ------------- | | | |  | |
