# Supplementary material for: Knowledge, attitude, and uptake of human papilloma virus vaccine and associated factors among female preparatory school students in Bahir Dar City, Amhara Region, Ethiopia
Source: PLoS One. 2022 Nov 21;17(11):e0276465. doi: 10.1371/journal.pone.0276465 (PMC9678319; doi:10.1371/journal.pone.0276465)
Supplement: Attachment — Submitted filename: PONE-D-22-21861.pdf [file pone.0276465.s004.pdf]

PLOS ONE

Knowledge, Attitude, and Uptake of Human Papilloma Virus Vaccine and Associated Factors Among Preparatory School Female Students in Bahir Dar City, Northwest, Ethiopia

--Manuscript Draft--

**Manuscript Number:**	PONE-D-22-21861
**Article Type:**	Research Article
**Full Title:**	Knowledge, Attitude, and Uptake of Human Papilloma Virus Vaccine and Associated Factors Among Preparatory School Female Students in Bahir Dar City, Northwest, Ethiopia
**Short Title:**	Knowledge, Attitude, and Uptake of Human Papilloma Virus Vaccine and Associated Factors , Ethiopia
**Corresponding Author:**	Habtamu Gebrehana Belay, MPH,MSc<br>Debre Tabor University<br>Debre Tabor, Amhara Region ETHIOPIA
**Keywords:**	Attitude, Human Papillomavirus Vaccine, Knowledge, Uptake, Ethiopia
**Abstract:**	**Background:** The human papillomavirus vaccine is one of the main preventative measures for cervical cancer. However, global vaccine uptake is low; the problem is particularly acute in low and middle-income countries. The purpose of this study is to assess female preparatory school students' knowledge, attitude, and uptake of the human papillomavirus vaccine and associated factors in Bahir Dar City, Northwest Ethiopia, in 2021.<br>**Methods:** - Institutional based cross-sectional study was conducted among 633 preparatory school female students in Bahir Dar city from March 1-30, 2021. Participants were selected using the multistage sampling technique. Data were collected using a structured self-administered questionnaire and entered into Epi-data and exported to SPSS for analysis. Binary and multivariable logistic regression analyses were done using an odds ratio with a 95% confidence interval. Finally-value < 0.05 was considered significant in multivariable analysis.<br>**Result:** The magnitudes of good knowledge about HPV vaccine, positive attitude, and uptake of human papillomavirus vaccine were 45.3 % (95% CI=41.6-49.4%), 58.1 % (95 %CI=54.4-61.9 %), and 16 % (95 %CI=13.2-19.5%), respectively. Having a history of sexual contact AOR=2.80 (95% CI=1.64-4.76), hearing about HPV infection AOR=1.59 (95 % CI=1.13-2.24), and having a positive attitude toward HPV vaccine AOR=1.46 (95 % CI=1.03-2.08) were significantly associated with knowledge about the HPV vaccine. Discussion of reproductive issues with family AOR=2.558 (95%CI=1.800-3.636), and having good knowledge about HPV vaccine AOR=3.571(95%CI=2.494-5.113) were associated with a positive attitude toward the HPV vaccine. Good knowledge AOR=2.36(95%CI=1.48 -3.76) and a positive attitude toward HPV vaccine AOR=2.87(95%CI=1.70-4.85) were strongly associated with HPV vaccine utilization<br>**Conclusion:** In this study, the magnitudes of good knowledge about the HPV vaccine, positive attitude, and uptake of the human papillomavirus vaccine were very low.
**Order of Authors:**	Etenesh Adela
	Eleni Adimassu
	Melash Belachew
	Habtamu Gebrehana Belay Gebrehana Belay
**Additional Information:**	
**Question**	**Response**
**Financial Disclosure**	no specific funding for this work

Powered by Editorial Manager® and ProduXion Manager® from Aries Systems Corporation

Enter a financial disclosure statement that describes the sources of funding for the work included in this submission. Review the submission guidelines for detailed requirements. View published research articles from *PLOS ONE* for specific examples.<br><br>This statement is required for submission and **will appear in the published article** if the submission is accepted. Please make sure it is accurate.<br><br>**Unfunded studies**<br>Enter: *The author(s) received no specific funding for this work.*<br><br>**Funded studies**<br>Enter a statement with the following details:<br>• Initials of the authors who received each award<br>• Grant numbers awarded to each author<br>• The full name of each funder<br>• URL of each funder website<br>• Did the sponsors or funders play any role in the study design, data collection and analysis, decision to publish, or preparation of the manuscript?<br>• **NO** - Include this sentence at the end of your statement: *The funders had no role in study design, data collection and analysis, decision to publish, or preparation of the manuscript.*<br>• **YES** - Specify the role(s) played.<br><br>* typeset	
**Competing Interests**<br><br>Use the instructions below to enter a competing interest statement for this submission. On behalf of all authors, disclose any competing interests that could be perceived to bias this work—acknowledging all financial support and any other relevant financial or non-financial competing interests.<br><br>This statement is required for submission and **will appear in the published article** if the submission is accepted. Please make	authors have declared that no competing interests exist.

sure it is accurate and that any funding sources listed in your Funding Information later in the submission form are also declared in your Financial Disclosure statement.<br><br>View published research articles from *PLOS ONE* for specific examples.<br><br>**NO authors have competing interests**<br><br>Enter: *The authors have declared that no competing interests exist.*<br><br>**Authors with competing interests**<br><br>Enter competing interest details beginning with this statement:<br><br>*I have read the journal's policy and the authors of this manuscript have the following competing interests: [insert competing interests here]*<br><br>* typeset	
**Ethics Statement**<br><br>Enter an ethics statement for this submission. This statement is required if the study involved:<br><br>• Human participants<br>• Human specimens or tissue<br>• Vertebrate animals or cephalopods<br>• Vertebrate embryos or tissues<br>• Field research<br><br>Write "N/A" if the submission does not require an ethics statement.<br><br>General guidance is provided below. Consult the submission guidelines for detailed instructions. **Make sure that all information entered here is included in the Methods section of the manuscript.**	Ethical clearance was obtained from the Institutional Review Board of Bahir Dar University College of Medicine and Health Sciences and a supporting letter was obtained from the Bahir Dar town education office. Permission was obtained from Bahir Dar tow preparatory schools to conduct the study. Furthermore, after providing appropriate information about the study and the right to refuse participation, each respondent provided verbal informed consent and assent. The parents and guardians of the minors verbally consented before the adolescent females were interviewed. Each school head ensured that participants gave their verbal approval. Respondents were informed that all the data obtained from them was kept confidential and anonymous. To ensure confidentiality, the names of respondents were replaced by code numbers.

Powered by Editorial Manager® and ProduXion Manager® from Aries Systems Corporation

**Format for specific study types**<br><br>**Human Subject Research (involving human participants and/or tissue)**<br>• Give the name of the institutional review board or ethics committee that approved the study<br>• Include the approval number and/or a statement indicating approval of this research<br>• Indicate the form of consent obtained (written/oral) or the reason that consent was not obtained (e.g. the data were analyzed anonymously)<br><br>**Animal Research (involving vertebrate animals, embryos or tissues)**<br>• Provide the name of the Institutional Animal Care and Use Committee (IACUC) or other relevant ethics board that reviewed the study protocol, and indicate whether they approved this research or granted a formal waiver of ethical approval<br>• Include an approval number if one was obtained<br>• If the study involved *non-human primates*, add *additional details* about animal welfare and steps taken to ameliorate suffering<br>• If anesthesia, euthanasia, or any kind of animal sacrifice is part of the study, include briefly which substances and/or methods were applied<br><br>**Field Research**<br><br>Include the following details if this study involves the collection of plant, animal, or other materials from a natural setting:<br>• Field permit number<br>• Name of the institution or relevant body that granted permission	
**Data Availability**<br><br>Authors are required to make all data underlying the findings described fully available, without restriction, and from the time of publication. PLOS allows rare exceptions to address legal and ethical concerns. See the PLOS Data Policy and FAQ for detailed information.	Yes - all data are fully available without restriction

A Data Availability Statement describing where the data can be found is required at submission. Your answers to this question constitute the Data Availability Statement and **will be published in the article**, if accepted.<br><br>**Important:** Stating 'data available on request from the author' is not sufficient. If your data are only available upon request, select 'No' for the first question and explain your exceptional situation in the text box.<br><br>Do the authors confirm that all data underlying the findings described in their manuscript are fully available without restriction?	
**Describe where the data may be found in full sentences. If you are copying our sample text, replace any instances of XXX with the appropriate details.**<br><br>• If the data are **held or will be held in a public repository**, include URLs, accession numbers or DOIs. If this information will only be available after acceptance, indicate this by ticking the box below. For example: *All XXX files are available from the XXX database (accession number(s) XXX, XXX.).*<br>• If the data are all contained **within the manuscript and/or Supporting Information files**, enter the following: *All relevant data are within the manuscript and its Supporting Information files.*<br>• If neither of these applies but you are able to provide **details of access elsewhere**, with or without limitations, please do so. For example:<br><br>*Data cannot be shared publicly because of [XXX]. Data are available from the XXX Institutional Data Access / Ethics Committee (contact via XXX) for researchers who meet the criteria for access to confidential data.*<br><br>*The data underlying the results presented in the study are available from (include the name of the third party*	HPV

and contact information or URL).<br>• This text is appropriate if the data are owned by a third party and authors do not have permission to share the data.<br><br>* typeset	
Additional data availability information:	Tick here if the URLs/accession numbers/DOIs will be available only after acceptance of the manuscript for publication so that we can ensure their inclusion before publication.

Powered by Editorial Manager® and ProduXion Manager® from Aries Systems Corporation

Manuscript Click here to access/download;Manuscript;Manuscript -- HPV.edited24.docx

Knowledge, Attitude, and Uptake of Human Papilloma Virus Vaccine and Associated Factors Among Preparatory School Female Students in Bahir Dar City, Northwest, Ethiopia

Etenesh Adela[1], Eleni Adimassu[2,] Melash Belachew[2,] Habtamu Gebrehana[3]

[1] Department of Midwifery, Debre Tabor Health Sciences College, Debre Tabor, Ethiopia.

[2]Bahir Dar University, College of Medicine and Health Sciences, School of Public Health, Department of Reproductive Health and Population Studies, Ethiopia.

[3]Department of Midwifery, College of Health Sciences, Debre Tabor University, Debre Tabor, Ethiopia.

Address:

[1*]Corresponding Author: Email: eteneshadela@gmail.com(EAL)

[2] eleniam16@gmail.com(EA)

[2]degualemu53@gmail.com(AD)

[3]hgebrehana@yahoo.com (HG)

Abstract

Background: The human papillomavirus vaccine is one of the main preventative measures for cervical cancer. However, global vaccine uptake is low; the problem is particularly acute in low and middle-income countries. The purpose of this study is to assess female preparatory school students' knowledge, attitude, and uptake of the human papillomavirus vaccine and associated factors in Bahir Dar City, Northwest Ethiopia, in 2021.

Methods: - Institutional based cross-sectional study was conducted among 633 preparatory school female students in Bahir Dar city from March 1-30, 2021. Participants were selected using the multistage sampling technique. Data were collected using a structured self-administered questionnaire and entered into Epi-data and exported to SPSS for analysis. Binary and multivariable logistic regression analyses were done using an odds ratio with a 95% confidence interval. Finally-value < 0.05 was considered significant in multivariable analysis.

Result: The magnitudes of good knowledge about HPV vaccine, positive attitude, and uptake of human papillomavirus vaccine were 45.3 % (95% CI=41.6-49.4%), 58.1 % (95 %CI=54.4-61.9 %), and 16 % (95 %CI=13.2-19.5%), respectively. Having a history of sexual contact AOR=2.80 (95% CI=1.64-4.76), hearing about HPV infection AOR=1.59 (95 % CI=1.13-2.24), and having a positive attitude toward HPV vaccine AOR=1.46 (95 % CI=1.03-2.08) were significantly associated with knowledge about the HPV vaccine. Discussion of reproductive issues with family AOR=2.558 (95%CI=1.800-3.636), and having good knowledge about HPV vaccine AOR=3.571(95%CI=2.494-5.113) were

associated with a positive attitude toward the HPV vaccine. Good knowledge AOR=2.36(95%CI=1.48 -3.76) and a positive attitude toward HPV vaccine AOR=2.87(95%CI=1.70-4.85) were strongly associated with HPV vaccine utilization

Conclusion: In this study, the magnitudes of good knowledge about the HPV vaccine, positive attitude, and uptake of the human papillomavirus vaccine were very low.

Keywords: Attitude, Human Papillomavirus Vaccine, Knowledge, Uptake, Ethiopia.

Introduction

Human Papilloma Virus (HPV) is a viral infection that passes through sexual skin-to-skin contact with someone who has it. It is one of the most common causes of sexually transmitted infections. It is estimated that 75% of sexually active people are infected with HPV during their lifetime[1]. More than 100 HPV genotypes are known. Of these, at least 13 genotypes are the cause of cervical cancer and genital and pharyngeal cancers. HPV types 16 and 18 cause about 70% of all cases of cervical cancer, vaginal, vulva, penis, and anus worldwide, and 90% of cases of genital warts are caused by HPV types 6 and 11[2, 3]. The vast majority of HPV infections are asymptomatic or sub-clinical, which has contributed to the rapid transmission and spread of the virus[4].

The human papillomavirus vaccine is one of the most effective ways to prevent HPV infections in women who have never been infected with HPV[5]. The HPV vaccine was first developed by the University of Queensland in Australia and the final form was made by Georgetown University medical center, the University of Rochester, and the United States (U.S). national cancer institute[6]. Food and drug agencies approved the first preventive HPV vaccine under the trade name of Gardasil, thereby 64 countries nationally implemented HPV immunization programs[7]. Two HPV vaccines are prequalified by the world health organization a bivalent and a quadrivalent vaccine and the vaccine can help prevent HPV infections. It can prevent more than 90% of these cancers[8].

One of the primary prevention strategies for HPV infection is vaccination. Two HPV vaccines (Gardasil and Cervarix) protect against the two strains of HPV types 16 and 18[9]. The quadrivalent vaccine is also highly efficacious in preventing genital warts, which are caused by

infection with HPV type 6 causes anogenital warts and HPV type 11 causes oropharyngeal cancer [10]. Gardasil works by stimulating the immune system to attack HPV types 6, 11, 16, and 18. Once Gardasil is administered, the body's immune system recognizes the viral proteins in Gardasil as foreign, and develops antibodies against them, thus providing immunity from future infections[11].

The vulnerability of young, sexually active women is recognized and steps are planned in many countries to implement HPV vaccination [12]. The world health organization recommends that the primary target group for HPV vaccination is girls aged 9–14 years and secondary target populations are females aged ≥ 15 years [8].In Ethiopia colorectal, cervical cancer, and breast cancer are respectable,5.7%,13.4%,30.2%[13], and cervical cancer is the 2nd leading cause of female cancer[14].

The HPV vaccine can prevent infection and cancer at an early age before any exposure to the virus. The HPV vaccine is recommended for everyone through the age of 26 years. Vaccination usually begins at 11 or 12 years of age, but can be given as early as 9 years of age. The decision to vaccinate should be individualized and discussed with the health care provider for older than 26 years of age[15]. The Advisory Committee on Immunization Practices now recommends a 2-dose series starting at 11 to 12 years[16]. Vaccinating girls before initiation of sexual activity is an important primary prevention intervention in a comprehensive cervical cancer prevention and control program. Exposure to HPV during adolescence and young adulthood may cause cervical cancer in later years. Because of this, Ethiopia had initially planned to introduce the HPV vaccine through a routine immunization program [17].

Previous research concentrated on cervical cancer screening; however, Ethiopia began using the HPV vaccine school-based method in December 2018. Studies on HPV vaccine awareness, attitudes, and uptake were scarce. As a result, the purpose of this study is to investigate HPV vaccine knowledge, attitude, and uptake and also its associated factors among preparatory school students in Bahir Dar, Ethiopia.

Methods and Materials

Study Area and period

An institutional-based cross-sectional study was conducted in Bahir Dar City among preparatory school students in the age group of 15-24 years from March 1-30, 2021. Bahir Dar is the capital city of the Amhara region, which is located 565 km away from Addis Ababa, the capital city of Ethiopia. The estimated total population of Bahir Dar City in 2019 was 445,084. Of this population, 222,097 (49.9 %) are males and 222,987 (50.1 %) are also females. Bahir Dar city had six government and five private preparatory schools.

Population

All-female night students registered in public institutions in Bahir Dar City for the academic year 2019 were the source population and those identified in the chosen public institution during the data collection period were the study population.

All female students enrolled in government and private preparatory schools for the 2021 academic year in Bahir Dar Town were the source population and those who were selected in those schools during the data collection period were the study population.

Inclusion and exclusion criteria

Female students registered in selected governmental and private preparatory schools in Bahir Dar town were included, while female students who had lived in the city for less than six months and those who were absent during the data collecting period after two separate visits were excluded.

Sample size determination

Considering the absence of previous data on specific study populations and knowledge, attitude, and uptake of HPV vaccination, the maximum sample size of 50% of prevalence was used in the calculation. The following assumptions were made: by considering the proportion of 50%, 95% confidence interval, and 5% margin of error. Using a design effect of 1.5 and a non-response rate of 10%, the total sample size was 633.

Sampling procedure

The multi-stage sampling technique was used to recruit participants for the study. First, preparatory schools were classified into public and private institutions, and then, using a simple random sampling process, three out of six governmental institutions and two out of four private schools were selected. The resulting sample size was then distributed proportionally to the selected schools. The rosters or name lists of students were utilized as a sampling frame to pick study subjects from each grade level. All students were sorted alphabetically. Finally, 633 students were selected using random computer generator software.

Operational definitions

Uptake of HPV vaccine: Refers to participants who had received at least 1 dose of HPV vaccine

Knowledge of HPV vaccine: Participants considered knowledgeable about the HPV vaccine when they answered more than the average number of 10 HPV vaccine knowledge questions

Attitudes toward the HPV vaccine: participants considered having a positive attitude towards the HPV vaccine when they scored above or equal to the average score of 12 attitude questions

Data collection procedure and techniques

The data was collected by using pretested and structured self-administered questionnaires after reviewing previous literature. The questionnaire was first developed in English and translated to the local language Amharic. Three diploma nurses who are familiar with the local language and customs were recruited as data collectors. Two-degree midwives were assigned supervisors for the data collectors and overall supervision also was made by the investigators.

Data Quality Assurance

The training was given to data collectors and supervisors for two days on data collection procedures, the content of the questionnaire, interview techniques, and confidentiality of the information obtained from the respondents regarding the knowledge, attitude, and uptake questions. Before conducting the main study, a pretest was carried out on 32(5%) of the sample size in Addis Zemen High school. The principal investigator and supervisor will conduct day-to-day on-site supervision during the whole period of data collection. At the end of each day, the questionnaires were reviewed and checked for completeness and accuracy by the supervisor and investigator. Data quality was ensured during collection, entry, and analysis. Before conducting the main study, a pretest was carried out on 32 (5%) of the sample. The principal investigator and supervisors conducted day-to-day on-site supervision during the whole period of data collection. At the end of each day, the questionnaires were reviewed and checked for completeness and accuracy by all the research team members, who undertook corrective discussion.

Data Processing and Analysis

Data were cleaned, coded, and entered by using Epi-Data version 4.6 and then exported to SPSS 21 for analysis. Descriptive statistics were carried out and frequency, tables, and graphs were used to present the descriptive results. Binary logistic regression analysis was executed by computing odds ratio (OR) with a 95% confidence interval to see the association between independent variables and dependent variables. On bivariable analysis, all independent variables associated with the dependent variable with $p \leq 0.25$ were entered into multivariable analysis, then the significant association will be identified based on $p < 0.05$ with 95% CI. Model fitness was checked by using Hosmer and Lemeshow goodness of fit.

Result

Socio-demographic characteristics

A total of 620 students were interviewed with a response rate of 98%. The mean age of study participants was 17 years (SD ±1.18). Almost 90% of participants were in the age range of 15-19 years. Three hundred ninety-eight (64.2%) of the participants were in grade 12 and more than two third of participants 482 (67.7%) were orthodox religious followers (Table1).

Source of information

Among the total participants, 257(41.5%) heard HPV vaccine on radio/television, 178(29%) hear from teachers, 128(20.6%) heard from the internet and 126(20%) heard from parents (Figure 1).

Knowledge about HPV Vaccine

Overall 281 (45.3%) of preparatory students have good knowledge about the human papillomavirus vaccine (figure 2).

More than half of respondents 320(51.6%) know the HPV vaccine prevents HPV infection and more than two third of participants 472(76.1%) knew that the HPV vaccine prevents cervical cancer. 361(58.2%) participants knew the HPV vaccine should be given before the first sexual intercourse and 291(46.9%) knew the HPV vaccine can be given to people who have had sex. More than half of respondents 334(53.9%) knew the recommended age for vaccination against HPV vaccine is the age range of 10-14 years and two third of participants 399(64.4%) did not know the recommended dose of HPV vaccine (Table 2).

Attitude towards HPV Vaccine

The overall positive attitude towards the HPV vaccine among respondents was 360 (58%). Two hundred eighty-two (47.1%) of the respondents strongly agree that the HPV vaccine is effective in preventing cervical cancer, 256(41.3%) respondents agree that they will take the vaccine because they feel at risk of getting HPV, 222(35.8%) believe that the side effects of the vaccine will not prevent from taking the vaccine. 223(34.0%) agreed that the HPV vaccine is better to be vaccinated before becoming sexually active and 212(34%) participants believe that more information on HPV and its vaccine will be needed before I take the vaccine (Table 3).

Uptake of HPV vaccine

The overall uptake of the HPV vaccine among respondents was 102(16%) and 518(84%) female students did not take the HPV vaccine. The reported reasons no vaccinated HPV vaccine was 79 (12.7%) respondents reasoned that due to not being informed by the health care providers, did not know about the safety of the vaccine 270(43.5%), did not know where the vaccine is got 93(15%) (Figure 3).

Factors associated with knowledge of the human papillomavirus vaccine

Multivariable logistic regression revealed that having ever sexual contact history, hearing about HPV infection and positive attitude about HPV were significantly associated with having good knowledge about the HPV vaccine. Students who have had a history of sexual contact increased almost 3 times the odds of knowledge about the HPV vaccine as compared to their counterparts [AOR=2.8, 95% CI= (1.64-4.76)]. Female students who heard about HPV infection increased 1.6 times the odds of knowledge about the HPV vaccine [AOR=1.59. 95% CI= (1.13-2.24)]. Having a positive attitude about the HPV vaccine increased 1.46 times the odds of knowledge about the HPV vaccine [AOR=1.46, 95%CI= (1.03-2.08] (Table 4).

Factors associated with attitudes toward the human papillomavirus vaccine

Female students who had discussed reproductive health issues with their partners increased 2.56 times the odds of attitude towards the HPV vaccine [AOR=2.56,95% CI= (1.80-3.64)] and students who had good awareness about the HPV vaccine increased 3.57 times the odds of HPV vaccine [AOR= 3.57, 95% CI= (2.49-5.11)] (Table 5).

Factors associated with uptake of human papillomavirus vaccine

Female students who have good knowledge about the HPV vaccine increased 2.36 times the odds of HPV vaccine utilization as compared to those who had poor knowledge about the HPV vaccine [AOR=2.36, 95% CI= (1.48-3.76)]. Students who have a positive attitude toward the HPV vaccine increased almost 3 times the odds of HPV vaccine utilization [AOR=2.87, 95% CI= (1.70-4.85)]. (Table 6).

Discussion

The primary goal of this study was to provide insight into the level of HPV vaccine knowledge, attitude, and uptake among school students, as well as to identify potential factors associated with HPV vaccine knowledge, attitude, and uptake.

This study revealed that the knowledge about of HPV vaccine among preparatory female students in the study area was found to be 45.3%. This finding is consistent with the findings of other studies in Debre Markos, Ethiopia(47.6%)[18] ,Indonesia (44.4%)[19], India (44%)[20] .However, this finding is relatively lower than that of study in Brazil at 51.79% [21], Italy at 56.3%[22], and Malaysia at 50.8%[23]. This study found that 58 % of the preparatory female students in the study area had a favorable attitude toward the HPV vaccine. This is consistent with a study conducted in Nigeria, which found 61.8% [24]. However, this result is less than those from India (69%) and Benin-City, Nigeria (61.8%)[25, 26].

This study revealed that only 16% of preparatory female students in Bahir Dar town were vaccinated with the HPV vaccine. This is slightly in line with studies done among female college students in mainland China at 11%[27], Mbale District, Uganda at 14%[28], and 16% of the female college students in Kenya had completed the full recommended two-dose schedule [29]. However, this result is significantly lower than the Ugandan study's 42.4%[30], Germany's 53%[31], and the United States' 62.4%[32]. This could be a result of the different sample sizes, study population, and strategies for the healthcare delivery system.

Teachers and the media (radio, television, and the internet) were the main sources of information about HPV vaccination, followed by parents and medical professionals. This is supported by research from Indonesia[33], Nigeria [34], and Turkey [35].

Students who had a history of sexual contact had heard of HPV infection and had a positive attitude toward the HPV vaccine were associated with HPV vaccine knowledge. Students who discuss reproductive issues with their partners and who are well-informed about HPV infection have significantly associated positive attitudes toward HPV vaccination. HPV vaccine knowledge and attitude are significantly associated with HPV vaccine uptake. Students who have a history of sexual contact are more likely to know about the PHV vaccine by 2.8 times. This is consistent with research conducted in Lagos State, Nigeria[36]. The possible reason might be due to students with a history of sexual activity who may fear STIs, HIV, and pregnancy-related issues. They also may be aware that the probability of getting HPV from sexual contact is high. they can learn about one of the vaccinations used to prevent HPV infection as a result.

Students ever heard about the HPV vaccine before the study increased 1.59 times the odds of knowledge of the HPV vaccine. This is in line with a study in Brazil[37]. The possible reason might be due to students who have heard about the HPV vaccine may read about it or ask others about its benefits. This increases their understanding of the HPV vaccine's disadvantages and side effects.

Students who had a positive attitude towards the HPV vaccine increased 1.46 time the odds of knowledge about the HPV vaccine. This is consistence with a study conducted in Debre Markos town [18]. This is might be due to the fact that individuals who have a positive attitude towards the vaccine may utilize it. Students' discussion of the reproductive issue with their parents increased the odds of attitude towards the HPV vaccine by 2.56 times. Parents who are transparent about sexuality with their young children have more robust communication, which is crucial for preventing risky sexual activities like early sexual initiation, unwanted pregnancy, and other reproductive health issues.

Having awareness about the HPV vaccine increased the attitude toward vaccination of HPV vaccine by 3.57 times. This is consistent with the study done in Debre Markos town, Ethiopia [18]. Having a satisfactory level of knowledge about the HPV vaccine increased their uptake of vaccination by 2.36 times. This is in line with research conducted in different parts of Ethiopia, including Ambo Town, Addia Ababa Town, and Gondar Town. [38-40].

HPV vaccination is influenced by practice factors, such as adequate health information systems that include provider or patient prompts for vaccine administration[41]. Supporting evidence indicates that adolescents are more likely to receive the HPV vaccine before they begin having sex when they are adequately informed about the vaccines and realize that the vaccine is safe, HPV-related diseases are serious, and adolescents are susceptible.[42]. Knowledge of HPV is important in how people think about their sexuality, protection, and prevention[43]. Having a positive attitude towards the HPV vaccine increased the uptake of the HPV vaccine by 2.87 times. This is a consistent study conducted in Benin-City, Nigeria **[25],** and Uganda **[44].** This may be explained by the fact that the majority of motivating/initiative factors in adolescents' practice is generated by their positive attitude.

Conclusion

The magnitudes of good knowledge about the HPV vaccine, positive attitude, and uptake of the human papillomavirus vaccine were all very low in the study. A history of sexual contact, hearing about HPV infection, and having a positive attitude toward the HPV vaccine were all significantly associated with knowledge of the HPV vaccine. Discussion of reproductive issues with family and knowledge of the HPV vaccine was linked to a favorable attitude toward the HPV vaccine. The

use of HPV vaccine was strongly associated with good knowledge and a favorable attitude toward the HPV vaccine.

Recommendations

We recommend the Ethiopian Ministry of Health conduct mass HPV vaccinations and community sensitizations to foster positive attitudes and ensure consistent vaccine availability at vaccination sites. For ease of access, the government should make an effort to add the HPV vaccine to the National Program on Immunization (NPI) schedule.

Abbreviations

ACIP: Advisory Committee on Immunization Practices; AOR: Adjusted Odd Ratio; CI: Confidence Interval; COR: Crude Odd Ratio; HPV: Human Papilloma Virus; OR: Odds Ratio

Declaration

Ethical approval and consent to participate

Ethical clearance was obtained from the Institutional Review Board of Bahir Dar University College of Medicine and Health Sciences and a supporting letter was obtained from the Bahir Dar town education office. Permission was obtained from Bahir Dar tow preparatory schools to conduct the study. Furthermore, after providing appropriate information about the study and the right to refuse participation, each respondent provided verbal informed consent and assent. The parents and guardians of the minors verbally consented before the adolescent females were interviewed. Each school head ensured that participants gave their verbal approval. Respondents were informed that all the data obtained from them was kept confidential and anonymous. To ensure confidentiality, the names of respondents were replaced by code numbers.

Consent for publication

Not applicable

Availability of Data and materials

The data used to support the findings of this study are available from the corresponding author upon formal request.

Competing interests

We declared that we did not have competing interests.

Funding

No fund was available

Authors' contributions

Etenesh Adela: Conceiving the research idea, writing the proposal, designing the study, supervising the data collection process, performing the statistical analysis, and writing the manuscript. Elenie Adimasu and Melash Belachew: reviewed and finalized the manuscript. Habtamu Gebrehana: Analysed and interpreted the data, analysis tools; Wrote the paper. All authors read and approved the final manuscript

Acknowledgment

We would like to acknowledge Bahirdar University College of Medicine and Health Sciences for their Ethical approval. Our deepest gratitude goes to our participants for their cooperation during the study as well as their permission to publish this article

Author Details

[1] Department of Midwifery, Debre Tabor Health Sciences College, Debre Tabor, Ethiopia.

[2]Bahir Dar University, College of Medicine and Health Sciences, School of Public Health, Department of Reproductive Health and Population Studies, Ethiopia.

[3]Department of Midwifery, College of Health Sciences, Debre Tabor University, Debre Tabor, Ethiopia.

Reference

1. *Weaver BA. Epidemiology and natural history of genital human papillomavirus infection. J Am Osteopath Assoc. 2006;106(3 suppl 1):S2-S8.*
2. *World Health Organization. (2011). Human papillomavirus (HPV). World Health Organization. Retrieved 15th February, 2013, from http://www.who.int/nuvi/hpv/en/.*

3. *Organization WH. Human papillomavirus vaccines: WHO position paper. Weekly Epidemiological Record= Relevé épidémiologique hebdomadaire. 2009;84(15):118-31.*
4. *Trottier H, Franco EL. The epidemiology of genital human papillomavirus infection. Vaccine. 2006;24(Supplement 1(0)):S4–S15.*
5. *Chan, S. S. C., Ng, B. H. Y., Lo, W. K., Cheung, T. H., & Chung, T. K. H. (2009). Adolescent girls' attitudes on human papillomavirus vaccination. Journal of Pediatric and Adolescent Gynecology, 22(2), 85–90.*
6. Agorastos, T., et al., *Safety of human papillomavirus (HPV) vaccines: a review of the international experience so far.* Vaccine, 2009. **27**(52): p. 7270-7281.
7. *Mac-Fiberesima G. Cervical Cancer Knowledge and Risk Behaviors Among Students of University of Port Harcourt, Nigeria 2016.*
8. *World Health Organization. Human papillomavirus vaccines:WHO Vaccine-Preventable Diseases Surveillance Standard, 2018.*
9. *Markowitz LE, Dunne EF, Saraiya M, Lawson HW, Chesson H, Unger ER. Quadrivalent human papillomavirus vaccine: Recommendations of the advisory committee on immunization practices (ACIP). Morb Mortal Wkly Rep 2007;56:1‑24.*
10. Bruni, L., et al., *Global estimates of human papillomavirus vaccination coverage by region and income level: a pooled analysis.* The Lancet Global Health, 2016. **4**(7): p. e453-e463.
11. Sockrider, M. and J.A. Krishnan, *How Vaccines Work to Prevent Infections.* American Journal of Respiratory and Critical Care Medicine, 2021. **203**(6): p. P16-P17.
12. *Chang, Y. T., Chen, Y. C., Hayter, M., & Lin, M. L . Menstrual and menarche experience among pubescent female students in Taiwan: Implications for health education and promotion practice. Journal of Clinical Nursing, 18 (14), 2040–2048.* 2009.
13. *HSDP-IV Woreda-Based Health Sector Annual Core plan, Federal Ministry of Health of Ethiopia (2013/2014).*
14. Lenahan, K., A.M. Stroup, and K. Demissie, *PMU39 CERVICAL CANCER PREVENTION THROUGH HUMAN PAPILLOMAVIRUS (HPV) VACCINATION: A CASE STUDY IN ETHIOPIA.* Value in Health, 2019. **22**: p. S715.
15. *CDC. Human papillomavirus vaccination: recommendations of the Advisory Committee on Immunization Practices (ACIP). MMWR Morb Mortal Wkly Rep. August 29, 2014.*
16. *Advisory Committee on Immunization Practices, Centers for Disease Control and Prevention (CDC. Immunization of health-care personnel: recommendations of the Advisory Committee on Immunization Practices (ACIP). .* MMWR. Recommendations and reports: Morbidity and mortality weekly report. Recommendations and reports. 2011 Nov 25;60(RR-7):1-45.
17. *Alene T, Atnafu A, Mekonnen ZA, Minyihun A. Acceptance of Human Papillomavirus Vaccination and Associated Factors Among Parents of Daughters in Gondar Town, Northwest Ethiopia. Cancer Management and Research. 2020;12:8519.*
18. Sinshaw, M.T., S. Berhe, and S.G. Ayele, *Knowledge and Attitude Towards Human Papillomavirus Vaccine and Associated Factors Among Mothers Who Have Eligible Daughters in Debre Markos Town, Northwest Ethiopia.* Infection and Drug Resistance, 2022. **15**: p. 781.
19. Endarti, D., et al., *Knowledge, perception, and acceptance of HPV vaccination and screening for cervical cancer among women in Yogyakarta Province, Indonesia.* Asian Pacific journal of cancer prevention: APJCP, 2018. **19**(4): p. 1105.
20. Rashid, S., S. Labani, and B.C. Das, *Knowledge, awareness and attitude on HPV, HPV vaccine and cervical cancer among the college students in India.* PloS one, 2016. **11**(11): p. e0166713.
21. Kops, N.L., et al., *Knowledge about HPV and vaccination among young adult men and women: Results of a national survey.* Papillomavirus Research, 2019. **7**: p. 123-128.

22. Trucchi C, A.D., Tafuri S, Sticchi L, Durando P, Costantino C, Varlese F, Di Silverio B, Bagnasco AM, Ansaldi F, et al. , *Assessment of knowledge, attitudes, and propensity towards HPV vaccine of young adult students in Italy. Vaccines. 2020;8(1):1–16.*
23. Jalani, F.F.M., et al., *Knowledge, attitude and practice of human papillomavirus (HPV) vaccination among secondary school students in rural areas of Negeri Sembilan, Malaysia.* International journal of collaborative research on internal medicine & public health, 2016.
24. Onowhakpor AO, O.V., Osagie OL, Odili CG. Community medicine and primary health care human papilloma virus vaccination : , *knowledge, attitude and uptake among female medical and dental students in a tertiary institution. J Community Med Prim Heal Care. 2016;28:101–08.*
25. Onowhakpor, A., et al., *Human Papilloma Virus vaccination: knowledge, attitude and uptake among female medical and dental students in a tertiary institution in Benin-City, Nigeria.* Journal of Community Medicine and Primary Health Care, 2016. **28**(2): p. 101-108.
26. Radhika, M. and M. Ahmed, *Awareness and knowledge of human papilloma virus (HPV) vaccine in prevention of cervical cancer among medical students.* International Journal of Reproduction, Contraception, Obstetrics and Gynecology, 2018. **7**(12): p. 5026-5031.
27. You, D., et al., *Human papillomavirus (HPV) vaccine uptake and the willingness to receive the HPV vaccination among female college students in China: a multicenter study.* Vaccines, 2020. **8**(1): p. 31.
28. Nabirye, J., et al., *Health system factors influencing uptake of Human Papilloma Virus (HPV) vaccine among adolescent girls 9-15 years in Mbale District, Uganda.* BMC Public Health, 2020. **20**(1): p. 1-11.
29. Karanja-Chege, C.M., *HPV vaccination in Kenya: The Challenges faced and Strategies to increase Uptake.* Frontiers in Public Health, 2022: p. 204.
30. Katagwa, V.N., et al., *Acceptability of human papilloma virus vaccination among primary school girls in Minakulu sub-county, northern Uganda.* European journal of cancer prevention: the official journal of the European Cancer Prevention Organisation (ECP), 2014. **23**(4): p. 294.
31. Remschmidt, C., et al., *Knowledge, attitude, and uptake related to human papillomavirus vaccination among young women in Germany recruited via a social media site.* Human vaccines & immunotherapeutics, 2014. **10**(9): p. 2527-2535.
32. Liu, G., L. Kong, and P. Du, *HPV vaccine completion and dose adherence among commercially insured females aged 9 through 26 years in the US.* Papillomavirus Research, 2016. **2**: p. 1-8.
33. Khatiwada M, K.C., Mediani HS, Delprat C, Van Hal G, Dochez C. , *Knowledge, Attitude and Acceptability of the Human Papilloma Virus Vaccine and Vaccination Among University Students in Indonesia. Frontiers in Public Health. 2021:607.*
34. Makwe CC, A.R., Odeyemi KA. , *Human papillomavirus (HPV) infection and vaccines: Knowledge, attitude and perception among female students at the University of Lagos, Lagos, Nigeria. .* J Epidemiol Glob Health. (2012) 2:199–206. doi: 10.1016/j.jegh.2012.11.001.
35. Uzunlar Ö, Ö.S., Ba¸ser E, Togrul C, Karaca M, Güngör T, *A survey on human papillomavirus awareness and acceptance of vaccination among nursing students in a tertiary hospital in Ankara, Turkey. .* Vaccine. (2013) 31:2191–5. doi: 10.1016/j.vaccine.2013.01.033
36. Oluwole EO, I.O., Adejimi AA, Balogun MR, Osanyin GE. , *Knowledge, attitude and uptake of human papillomavirus vaccination among female undergraduates in Lagos State, Nigeria. J Family Med Prim Care 2019;8:3627-33.*
37. Gattegno, M.V., et al., *A cross-sectional survey of parental attitudes towards Human papillomavirus vaccination exclusion categories in Brazil.* BMC International Health and Human Rights, 2019. **19**(1): p. 1-9.

38. Beyen, M.W.m., et al., *Human papillomavirus vaccination uptake and its associated factors among adolescent school girls in Ambo town, Oromia region, Ethiopia, 2020.* PloS one, 2022. **17**(7): p. e0271237.
39. Dereje N , A.A.A., Abera A e al., *Knowledge and acceptance of HPV vaccination and its associated factors among parents of daughters in Addis Ababa, Ethiopia: a community-based cross-sectional study.* Infect Agents Cancer, 2021. **2021**: p. 16-58.
40. Alene T , A.A., Mekonnen ZA , Minyihun A, *Acceptance of Human Papillomavirus Vaccination and Associated Factors Among Parents of Daughters in Gondar Town, Northwest Ethiopia.* Cancer Management and Research, 2020. **12**.
41. Gilkey, M.B. and A.-L. McRee, *Provider communication about HPV vaccination: a systematic review.* Human vaccines & immunotherapeutics, 2016. **12**(6): p. 1454-1468.
42. SOUSA P, T.A., Baracat EC, Sorpreso IC, de Abreu LC., *Knowledge and acceptance of HPV vaccine among adolescents, parents and health professionals: construct development for collection and database composition. .* Journal of human growth and development. 2018.
43. Contreras-González, R., et al., *Nivel de conocimientos en adolescentes sobre el virus del papiloma humano.* Enfermería universitaria, 2017. **14**(2): p. 104-110.
44. Kisaakye, E., et al., *Level and factors associated with uptake of human papillomavirus infection vaccine among female adolescents in Lira District, Uganda.* Pan African Medical Journal, 2018. **31**(1).

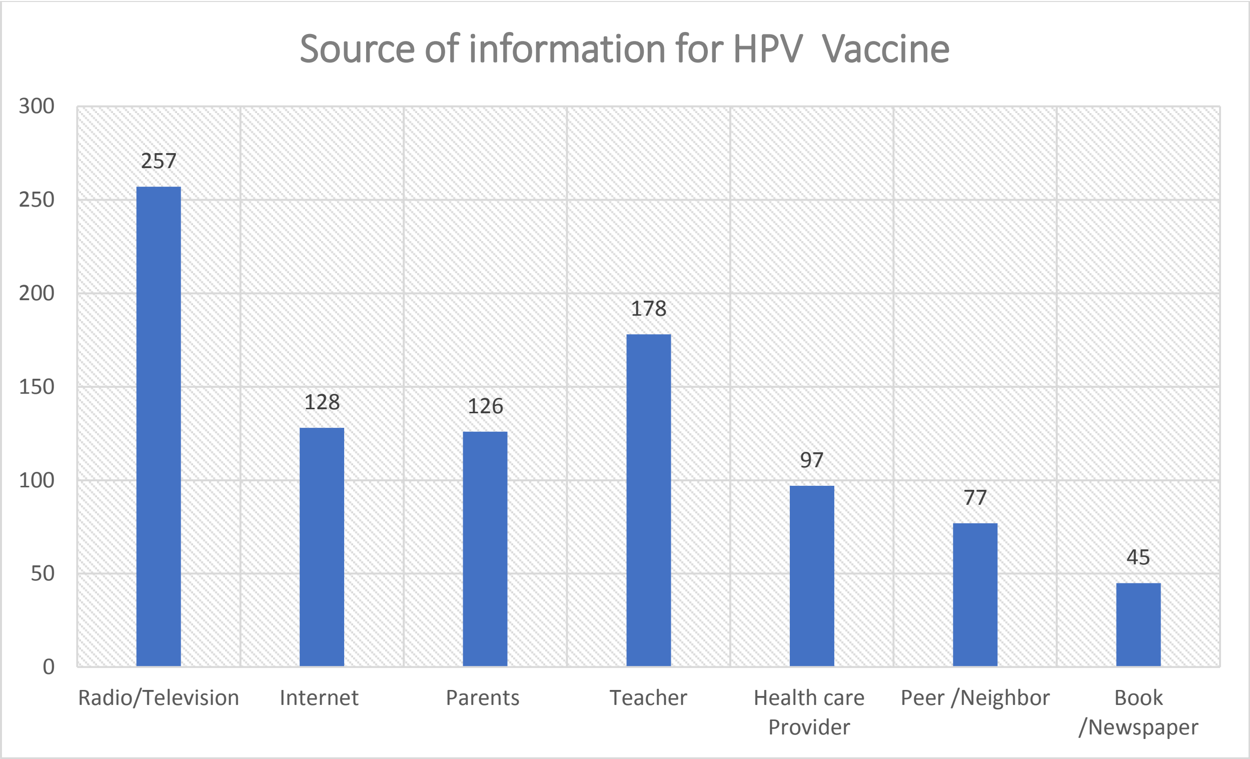

Figure 1: Source of information about HPV vaccine, Bahir Dar town, Ethiopia,2021

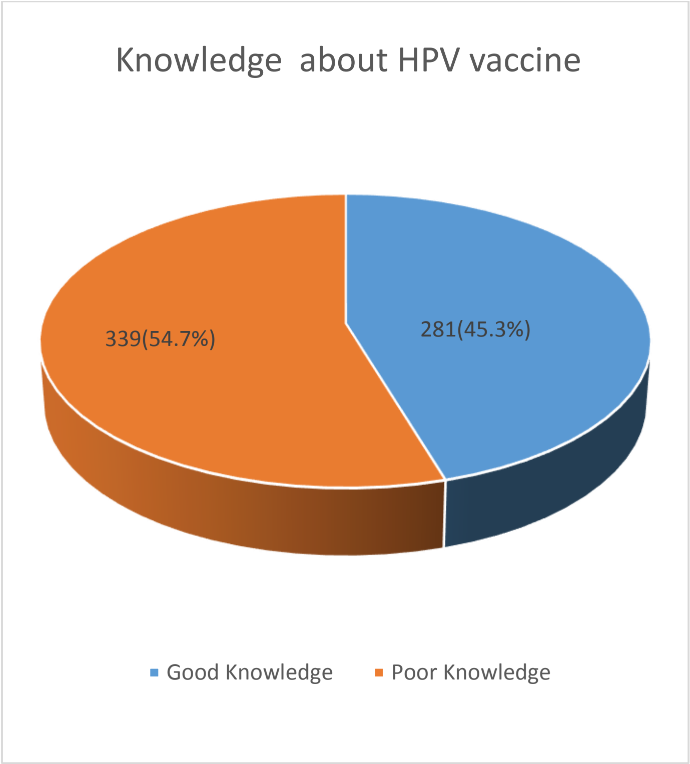

Figure 2: Knowledge of respondents about HPV vaccine, Bahir Dar town, Ethiopia,2021

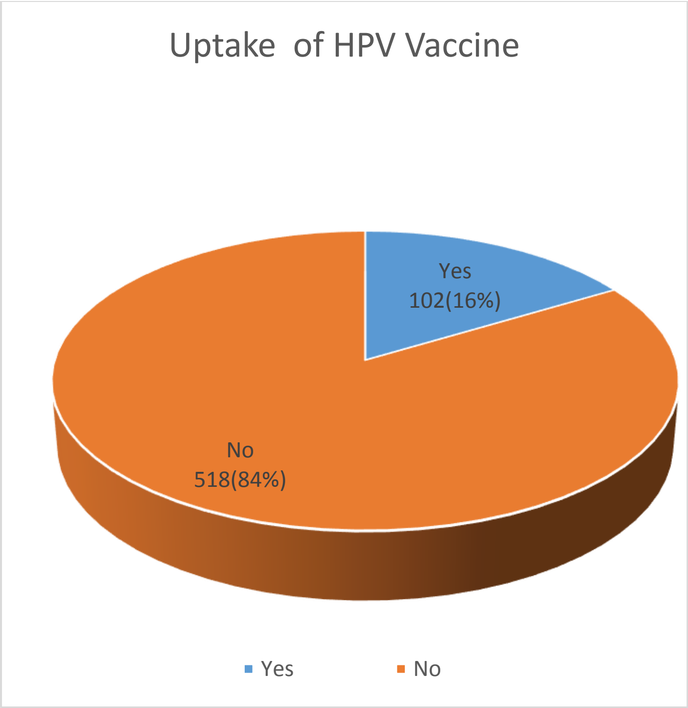

Figure 3: Uptake of HPV vaccine among preparatory school students, Bahir Dra town, Ethiopia ,2021

Click here to access/download;Figure;HPV-_Tables.docx

Table1: Socia-demographic characteristics of preparatory students in Bahir Dar Town, Ethiopia

Variables	Category	Frequency	Percent
Age	15-19	555	89.5%
	20-24	65	10.5%
Religion	Orthodox	482	67.7%
	Muslim	79	12.7%
	Catholic	32	5.2%
	Protestant	27	4.4%
Grade level	Grade 11	222	35.8%
	Grade 12	398	64.2%
Parents educational status	Informal education (can read and write)	84	13. 5%
	Primary and secondary	349	56.3 %
	Diploma and above	187	30 .2 %
Parents occupation	House wife	179	29%
	Employed	240	39%
	Merchant	163	26 %
	Others	38	6%
Average family monthly income	<4000	85	13.7%
	4000-7000	336	54.2%
	>7000	199	32.1%

Table 2: Knowledge towards HPV vaccine among preparatory school students in Bahir Dar town, Ethiopia, 2021

**Knowledge items**	**Category**	**Frequency**	**%**
Does human papilloma virus vaccine prevent human papilloma virus infection?	Yes	320	51.6%
	No	300	48.4%
Does human papilloma virus vaccine prevent cervical cancer?	Yes	472	76.1%
	No	148	23.9%
Is the human papillomavirus vaccine used to prevent genital warts?	Yes	320	51.6%
	No	300	48.4%
Should a human papilloma virus vaccine be given prior to the first sexual intercourse?	Yes	361	58.2%
	No	259	41.8%
Can human papilloma virus vaccine be given to people who have had sex?	Yes	291	46.9%
	No	329	53.1%
Can human papillomavirus vaccine be given to a woman who is already infected with HPV?	Yes	285	46.0%
	No	335	54.0%
What age range is recommended for vaccination against human papillomavirus infection?	10-14years	334	53.9%
	No	286	46.1%
How many doses are recommended for a human papillomavirus vaccine?	Two does	221	35.6%
	No	399	64.4%
Did you know the schedule for a human papillomavirus vaccine?	6 month-1year	217	35.0%
	No	403	65.0%
Did you know the site of vaccine for HPV vac	Arm	195	31.5%
	No	425	68.5%

Table 3: Attitude about HPV vaccine among preparatory school female students, Bahir Dar town, Ethiopia, 2021.

Attitude items	Category	frequency	Percent
HPV vaccine is effective in preventing cervical cancer	Strongly agree	292	47.1%
	Agree	186	39.0%
	Neutral	67	10.8%
	Disagree	52	8.4%
	Strongly disagree	23	3.7%
I will take the vaccine because I feel at risk of getting HPV	Strongly agree	152	24.5%
	Agree	256	41.3%
	Neutral	112	18.1%
	Disagree	72	11.6%
	Strongly disagree	28	4.5%
Person who has only one sexual partner can protect from HPV infection	Strongly agree	147	23.7%
	Agree	231	37.3%
	Neutral	146	23.5%
	Disagree	76	12.5%
	Strongly disagree	20	3,2%
It's not necessary to get the human papilloma virus vaccination	Strongly agree	139	22.4%
	Agree	38	13.4%
	Neutral	94	15.2%
	Disagree	217	35.0%
	Strongly disagree	87	14.0%
I believe that the side effects of the vaccine. Reasonable and will not prevent me from taking the vaccine.	Strongly agree	165	26.6%
	Agree	222	35.8%
	Neutral	131	21.1%
	Disagree	73	11.8%
	Strongly disagree	24	3.9%
I feel it is better to be vaccinated before becoming sexually active	Strongly agree	217	35%
	Agree	223	34.0%
	Neutral	121	19.5%
	Disagree	50	8 %
	Strongly disagree	9	1.5%
More information on HPV and its vaccine will be needed before I take the vaccine.	Strongly agree	260	42%
	Agree	212	34%
	Neutral	101	16%
	Disagree	36	6%
	Strongly disagree	11	2%
Human papilloma virus vaccine may have long negative effect	Strongly agree	147	23.7%

	Agree	147	23,7%
	Neutral	157	26.9%
	Disagree	122	18.7%
	Strongly disagree	37	6.0%
I feel only sexually active ladies should get the vaccine	Strongly agree	127	20.5%
	Agree	111	17.9%
	Neutral	146	23.5%
	Disagree	154	24.8%
	Strongly disagree	82	13.2%
Do you think your family allow to vaccinate	Strongly agree	141	22.7%
	Agree	113	18.2%
	Neutral	164	26.5%
	Disagree	143	23.1%
	Strongly disagree	59	9.5%
Education on HPV vaccine should be implemented at school	Strongly agree	243	39.2%
	Agree	226	36.5%
	Neutral	79	12.7%
	Disagree	54	8.7%
	Strongly disagree	18	2.9%
HPV vaccination should be included on the National Program on immunization.	Strongly agree	306	49.4%
	Agree	156	25.2%
	Neutral	87	14.0%
	Disagree	45	7.3%
	Strongly disagree	26	4.2%

Table 4: Factors associated with knowledge about HPV vaccine among preparatory school female students, Bahir Dar town, Ethiopia, 2021.

Variables	Knowledge on HPV vaccine		COR (95% CI)	AOR (95%CI)
	Good	Poor		
**Had ever sexual contact history**				
Yes	93	45	3.23(2.17-4.82)	2.80(1.64-4.76)
No	188	284	1	1
**Heard about HPV infection**				
Yes	160	155	1.57(1.14-2.16)	1.59(1.13-2.24)
No	121	184	1	1
**Attitude about HPV vaccine**				
Positive attitude	178	182	1.49(1.08-2.05)	1.46(1.03-2.08)
Negative attitude	103	157	1	1

Table 5: Factors associated with attitude about HPV vaccine among preparatory school female students, Bahir Dar town, Ethiopia, 2021.

**Variables**	**Attitude towards HPV vaccine**		**COR (95%CI)**	**AOR (95%CI)**
	**Positive Attitude**	**Poor Attitude**		
**Discussion of RH issues with family**				
Yes	147	78	3.22(2.25-4.62)	2.56(1.80-3.64)
No	213	182	1	1
**Awareness about HPV Vaccine**				
Good knowledge	210	71	3.73(2.62-5.26)	3.57(2.49-5.11)
Poor knowledge	150	189	1	1

Table 6: Factors associated with uptake of HPV vaccine among preparatory school female students, Bahir Dar town, Ethiopia, 2021

Variables	Uptake about HPV vaccine		COR	AOR
	Yes	No		
**Knowledge about HPV vaccine**				
Good knowledge	65	216	2.46(1.58-3.82)	2.36(1.48 -3.76)
Poor knowledge	37	302	1	1
**Attitude about HPV vaccine**				
Positive attitude	79	281	3.17(1.76-4.76)	2.87(1.70-4.85)
Poor attitude	23	237	1	1
