# Supplementary material for: Knowledge, attitude, and uptake of human papilloma virus vaccine and associated factors among female preparatory school students in Bahir Dar City, Amhara Region, Ethiopia
Source: PLoS One. 2022 Nov 21;17(11):e0276465. doi: 10.1371/journal.pone.0276465 (PMC9678319; doi:10.1371/journal.pone.0276465)
Supplement: Attachment — Submitted filename: Review Comments to the Author-HPV.docx [file pone.0276465.s005.docx]

**Review Comments to the Author**

Please use the space provided to explain your answers to the questions above. You may also include additional comments for the author, including concerns about dual publication, research ethics, or publication ethics. (Please upload your review as an attachment if it exceeds 20,000 characters)

Reviewer #1: The author needs to review the spelling and tenses in the manuscript. Also, in the result section, the author needs to rewrite it for clarity. I have highlighted and written comments on areas that need attention in the manuscript.

- **Response:** Comments have been accepted, and grammatical and spelling errors have been corrected.

Reviewer #2:

1. bearing in mind the several problems have been identified with the HL test. For example, it doesn’t take overfitting into account and tends to have low power, what informed the choice of Hosmer and Lemeshow goodness of fit in your methodology

- **Response:** Testing goodness of fit is an important step in evaluating a statistical model. For binary logistic regression models, the Hosmer–Lemeshow goodness-of-fit test is often used. The test assesses whether or not the observed event rates match expected event rates in subgroups of the model population

2. Is it descriptive or analytical cross-sectional study

- **Response:** This study is descriptive cross-sectional study. In descriptive cross-sectional study, the investigator measures the outcome and the exposures in the study participants at the same time. So that we assessed the prevalence of knowledge, attitude and uptake of HPV vaccination and its associated factors at the same time.

3. It’s an emerging fact that a single-dose Human Papillomavirus (HPV) vaccine delivers solid protection against HPV, the virus that causes cervical cancer, that is comparable to 2-dose schedules, NO MENTION OF IT IN YOUR MANUSCRIPT

- **Response:** we correct is accordingly. Only 102 (16%) of respondents had received the HPV vaccine, with one-thirds (33%) having completed the vaccine doses

**N.**B. There are no ethical or legal restrictions on sharing a de-identified data set. So that we shared the data in the under heading of supporting information.
